# Aspirin and the metabolic hallmark of cancer: novel therapeutic opportunities for colorectal cancer

**DOI:** 10.37349/etat.2023.00155

**Published:** 2023-08-28

**Authors:** Ashley J. Hoskin, Amy K. Holt, Danny N. Legge, Tracey J. Collard, Ann C. Williams, Emma E. Vincent

**Affiliations:** University of Turin, Italy; ^1^Department of Cellular and Molecular Medicine, Biomedical Sciences Building, University of Bristol, BS8 1TW Bristol, UK; ^2^Department of Translational Health Sciences, Dorothy Hodgkin Building, University of Bristol, BS1 3NY Bristol, UK; ^3^MRC Integrative Epidemiology Unit, Oakfield House, University of Bristol, BS8 2BN Bristol, UK

**Keywords:** Aspirin, cancer metabolism, metabolic hallmark, colorectal cancer, cancer prevention, nonsteroidal anti-inflammatory drugs, adjuvant for therapy, anti-tumour

## Abstract

Aspirin is a well-known nonsteroidal anti-inflammatory drug (NSAID) that has a recognized role in cancer prevention as well as evidence to support its use as an adjuvant for cancer treatment. Importantly there has been an increasing number of studies contributing to the mechanistic understanding of aspirins’ anti-tumour effects and these studies continue to inform the potential clinical use of aspirin for both the prevention and treatment of cancer. This review focuses on the emerging role of aspirin as a regulator of metabolic reprogramming, an essential “hallmark of cancer” required to support the increased demand for biosynthetic intermediates needed for sustained proliferation. Cancer cells frequently undergo metabolic rewiring driven by oncogenic pathways such as hypoxia-inducible factor (HIF), wingless-related integration site (Wnt), mammalian target of rapamycin (mTOR), and nuclear factor kappa light chain enhancer of activated B cells (NF-κB), which supports the increased proliferative rate as tumours develop and progress. Reviewed here, cellular metabolic reprogramming has been identified as a key mechanism of action of aspirin and include the regulation of key metabolic drivers, the regulation of enzymes involved in glycolysis and glutaminolysis, and altered nutrient utilisation upon aspirin exposure. Importantly, as aspirin treatment exposes metabolic vulnerabilities in tumour cells, there is an opportunity for the use of aspirin in combination with specific metabolic inhibitors in particular, glutaminase (GLS) inhibitors currently in clinical trials such as telaglenastat (CB-839) and IACS-6274 for the treatment of colorectal and potentially other cancers. The increasing evidence that aspirin impacts metabolism in cancer cells suggests that aspirin could provide a simple, relatively safe, and cost-effective way to target this important hallmark of cancer. Excitingly, this review highlights a potential new role for aspirin in improving the efficacy of a new generation of metabolic inhibitors currently undergoing clinical investigation.

## Introduction

Colorectal cancer (CRC) is a major disease and in 2020, there was an estimated 1.93 million incidences globally [1], with a worrying increase in incidence in the younger population [2–4]. As CRC is multistep process, early detection and effective prevention remain the most promising way to reduce CRC-associated mortality. Aspirin has a long-established association with a reduced risk of CRC [5–12]. However, the optimal dose and duration for effective prevention remains up for debate and more recent studies suggest risks may outweigh benefits for older individuals [6, 13–15]. Importantly, the potential therapeutic use of aspirin is also being explored and significant research is being undertaken to define a role for aspirin in CRC treatment. To better use this widely available, cost-effective drug for the management of CRC, it is important to understand its mechanism of action.

## Aspirin and CRC

Aspirin (also known as acetylsalicylic acid) is a nonsteroidal anti-inflammatory drug (NSAID) used as an analgesic and anti-inflammatory agent, often given to patients at high risk of cardiovascular disease [16, 17]. Like other NSAIDs, aspirin inhibits cyclooxygenases (COXs, COX1 and COX2), enzymes responsible for the generation of prostanoids from arachidonic acid [18, 19]. COX1 is constitutively expressed and important for the production of the thromboxane (TX), TXA2, which is involved in platelet aggregation and vasoconstriction. By contrast, COX2 is inducible and is often expressed in inflammatory and hypoxic conditions where it is involved in prostaglandin E_2_ (PGE_2_) synthesis. Although the COX pathway is a well-established target of aspirin, recent evidence has highlighted additional COX-independent actions which have become increasingly important in explaining the efficacy of aspirin for cancer prevention and therapy [20–22].

CRC is the fourth most common cancer in the UK and accounts for 10% of all cancer mortalities, placing it the second most common cause of cancer death in the UK [4]. Evidence of the association between aspirin use and reduced CRC incidence is substantial [5–12]. Evidence first arose from observational studies and meta-analyses of randomised control trials (RCTs) for the prevention of vascular disease and is the subject of many excellent reviews [23, 24]. A meta-analysis of all observational studies up to 2019 indicated a dose-dependent risk reduction of 10–35% (75–100 mg/day conveys 10% risk reduction and 325 mg/day conveys 35% risk reduction) and concludes that aspirin use is associated with a ≥ 25% CRC risk reduction in forty-five observational studies [25]. Based on this evidence, the United States Preventative Task Force (USPTF) recommends low-dose aspirin use for the prevention of CRC in 50–59 years old adults [26]. In the UK, aspirin is recommended for patients with Lynch syndrome, a familial condition characterised by germline mutations in DNA mismatch repair genes, most commonly MutS homolog 2 (MSH2) and MutL homolog 1 (MLH1) [27].

Although less well established, there is also evidence for the use of aspirin as an adjuvant therapy. Rothwell et al. [28] have shown that aspirin ranging from 75–1,200 mg/day is associated with a 21% reduction in mortality from any cancer. In the context of CRC, a cohort study identified doses of 75–300 mg/day of aspirin post-diagnosis to be protective against CRC-associated mortality, this was significant at the lowest dose (75 mg/day) [29]. Several other studies also associate aspirin use with decreased CRC mortality [30–34]. A commonality between these studies is that to observe reduced mortality, aspirin must be administered post-diagnosis rather than pre-diagnosis. The effects of aspirin on mortality are seen after a period of ~5 years [35], which is thought to be due to aspirin reducing the risk of metastasis. A review of five RCTs found a reduced risk of metastasis with daily aspirin use but this only included patients who started taking aspirin post-diagnosis [36]. Research into the use of aspirin as an adjuvant therapy is ongoing; the add-aspirin trial is designed to investigate aspirin use and colorectal survival (alongside breast, prostate, stomach, and oesophageal cancer) and includes doses ranging between 100–300 mg/day (tertiary prevention) [37].

One disadvantage of aspirin is that it carries a risk of gastrointestinal (GI) bleeding, with a low dose of aspirin use associated with a risk of 2–4 GI bleeds per 1,000 patients. However, this risk depends on the patient’s age, gender, and history of GI ulcers [38]. The ASPirin in Reducing Events in the Elderly (ASPREE) trial concluded that aspirin increased the risk of major hemorrhage in adults > 70 years old although this risk was age-dependent [14]. The absolute risk of serious GI bleeding was 0.25% for a 70-year-old with no additional contributing factors. However, this increased to a 5% risk for an 80-year-old with multiple risk factors (smoking, hypertension, and previous NSAID use) [14]. If aspirin is to be used routinely in a cancer setting, it is crucial to identify optimal treatment regimens and patient subgroups who would benefit from aspirin use.

**Figure 1:**
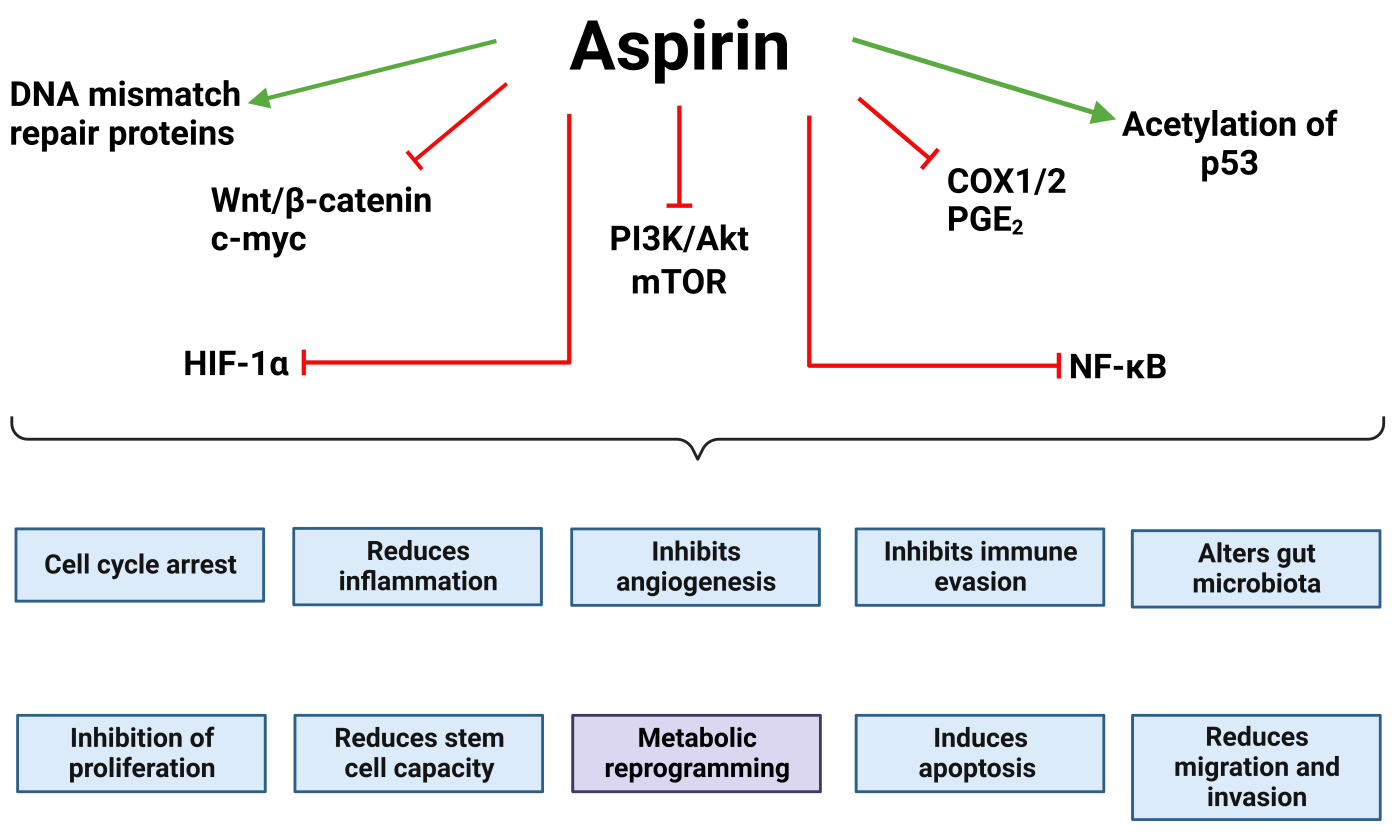
Oncogenic drivers regulated by aspirin. Aspirin has been shown to inhibit or reduce the activity of key oncogenic drivers, many of which inhibit the “hallmarks of cancer” [41]. This includes Wnt signalling and its downstream effectors, HIF-1α, the PI3K/Akt/mTOR signalling axis, NF-κB signalling, and COX1/2 and its downstream effectors. Aspirin has also been shown to increase the acetylation and stability of p53 leading to cell cycle arrest and apoptosis. Together this demonstrates how aspirin impacts a number of the hallmarks of cancer. The green arrows indicate that aspirin promotes that effect/pathway and the red blunt arrows indicate that aspirin inhibits it. The figure was created with BioRender.com. c-myc: cellular myc; Akt: protein kinase B or Akt

It is important to understand the mechanism by which aspirin is having anti-tumour effects, potentially opening the door to further “aspirin-related drugs” for CRC prevention and therapy. There has been an increasing number of studies contributing to the mechanistic understanding of aspirin’s anti-tumour effects in CRC including regulation of wingless-related integration site (Wnt), nuclear factor kappa light chain enhancer of activated B cells (NF-κB), hypoxia-inducible factor-1α (HIF-1α), mammalian target of rapamycin (mTOR), and phosphatidylinositol 3-kinase (PI3K) pathways which is the subject of excellent reviews (**Figure 1**) [20, 22, 39, 40]. More recently a role for aspirin in metabolic reprogramming of cancer cells has begun to emerge, which is the focus of this review.

## Aspirin targets metabolic reprogramming in CRC

**Figure 2:**
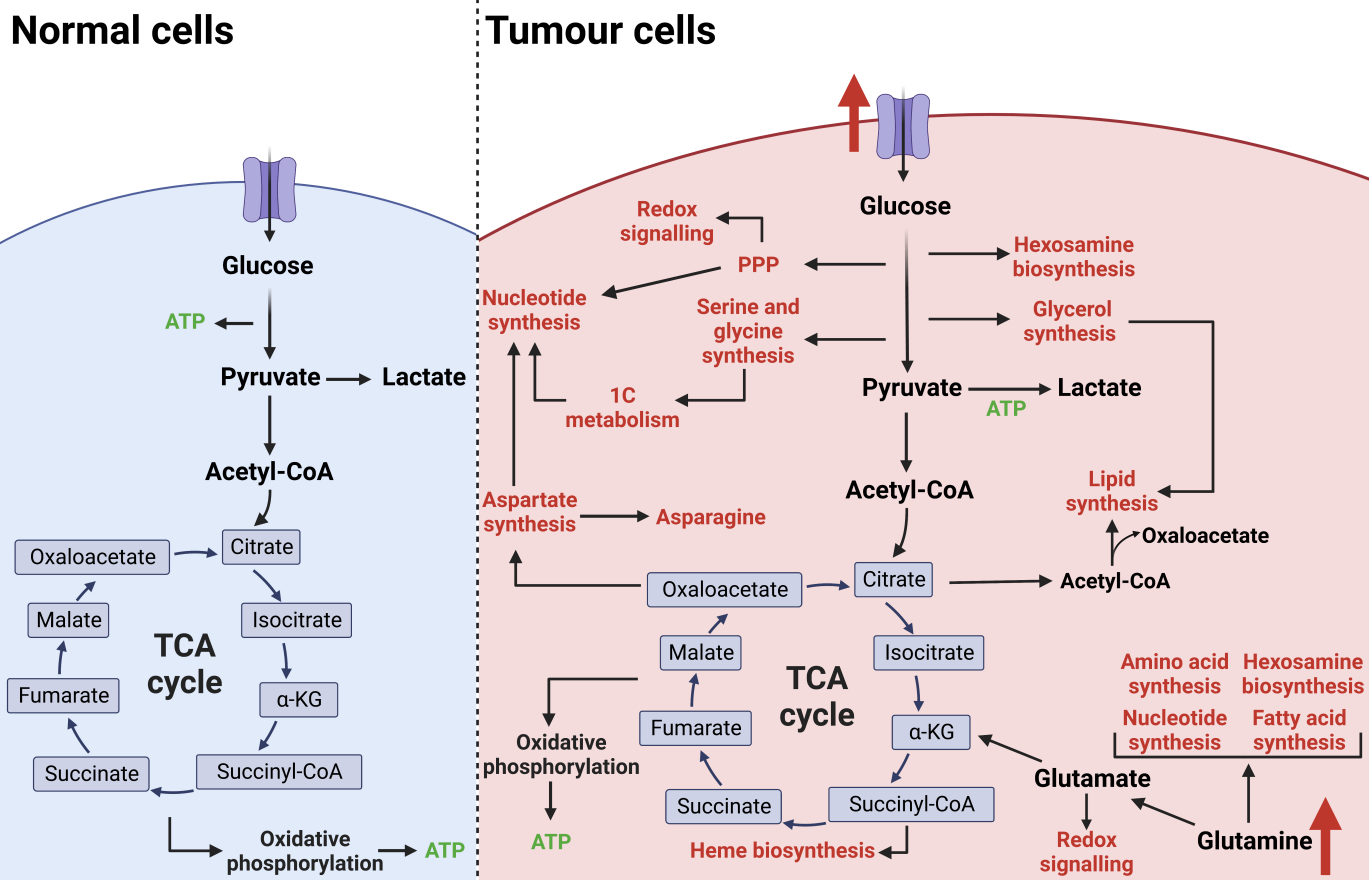
Metabolic reprogramming of tumour cells. In non-malignant cells under aerobic conditions, glucose is utilised as the primary fuel source to generate ATP via glycolysis, the TCA cycle, and oxidative phosphorylation. Cancer cells rewire metabolic pathways to favour biomass production over ATP generation which is required to support an increased proliferative rate. Increased nutrient uptake is primarily used to fuel anabolic branchpoints from glycolysis and the TCA cycle. This reprogramming of cellular metabolism supports tumour development and progression. The figure was created with BioRender.com. acetyl-CoA: acetyl-coenzyme A; succiyl-CoA: succiyl-coenzyme A; TCA: tricarboxylic acid; 1C: one carbon; PPP: pentose phosphate pathway

The reprogramming of cellular metabolism is an established hallmark of cancer and is essential to support the increased demand for biosynthetic intermediates required for sustained proliferation [41]. In non-malignant cells, nutrient-sourced carbons are predominantly used to generate adenosine triphosphate (ATP). However, tumour cells increase the import of nutrients from the tumour microenvironment and reprogramme metabolic pathways, primarily to fuel biosynthetic processes (**Figure 2**). Shifting to anabolic metabolism allows biomass accumulation through nucleotide, amino acid, and lipid synthesis. Oncogenic pathways alongside mutations in metabolic enzymes can rewire nutrient consumption and utilisation to meet the bioenergetic and biosynthetic needs of the tumour cell. For example, the receptor tyrosine kinase (RTK)-PI3K-Akt pathway commonly drives the expression of glucose transporter 1 (GLUT1) and subsequently increased glucose uptake for tumour biomass production [42]. CRC cells exhibit common features of aberrant metabolism such as increased glycolytic flux (the Warburg effect) and glutamine utilisation [43–45]. The reprogramming of CRC cell metabolism supports tumour development and the switch to an increased glycolytic rate has been shown to occur early in CRC progression [46–48]. Therefore, targeting cancer metabolism is an attractive therapeutic approach. Antimetabolites such as 5-fluorouracil (5-FU) are routinely used as chemotherapy; these antimetabolites target the increased demand for nucleotide synthesis [49]. However, targeting proliferative metabolism in general often leaves an inadequate therapeutic window as non-malignant rapidly proliferating cells rely on a similar metabolic programme which results in toxicity. To combat this, there is a focus on targeting specific metabolic dependencies adopted by malignant cells for novel therapeutic strategies. In recent years, specific metabolic inhibitors have gained momentum for cancer therapy such as ivosidenib and enasidenib for relapsed/refractory IDH-mutated acute myeloid leukaemia [50, 51].

Glutamine dependency of tumour cells is an attractive therapeutic target, glutamine analogues reached clinical trials many years ago but were discarded due to lack of efficacy [52]. This is commonly seen when using metabolic inhibitors as single agents due to the ability of cancer cells to overcome metabolic perturbation by inducing compensatory pathways to survive. For example, upon glutaminase (GLS) inhibition in pancreatic ductal adenocarcinoma (PDAC) cells, the upregulation of pyruvate carboxylase (PC), activation of lipid biosynthetic pathways, and alternative pathways involved in glutamate production allowed cells to adapt and regain proliferation [53]. It is extremely important to analyse and understand the compensatory mechanisms induced by metabolic inhibition, as this gives rise to the possibility of combination therapies. Targeting metabolic enzymes/pathways in combination therapies restricts the adaptive metabolic network and the ability to adopt alternative pathways which are needed to circumvent metabolic inhibition. Here we propose that, through its action on cancer cell metabolism, aspirin could provide a simple, relatively safe, and cost-effective way to target this important hallmark of cancer and potentially be used as adjuvant therapy to improve the efficacy of metabolic inhibitors already in clinical trials.

There have been a number of studies investigating the role of aspirin in metabolic rewiring in CRC. A metabolomics study on human colon tissue taken from patients given 81 mg aspirin a day found the main metabolic processes associated with aspirin treatment were energy, nucleotide, and amino acid metabolism [54]. Importantly, 81 mg of aspirin a day was associated with a decrease in adenoma risk suggesting the regulation of these processes is key to the chemopreventative effect of aspirin. Mechanistically, aspirin has been found to acetylate several metabolic enzymes, 14 lysine residues were found to be acetylated by aspirin on the glucose-6-phosphate dehydrogenase (G6PD) protein [55]. G6PD is a key enzyme in the pentose phosphate pathway, a biosynthetic branchpoint from glycolysis generating ribose-5-phosphate, which is essential for nucleotide synthesis and nicotinamide adenine dinucleotide phosphate (NADPH), an important reducing agent for protection against reactive oxygen species (ROS). The acetylation of G6PD by aspirin markedly decreased its enzymatic activity, suggested to contribute to reduced synthesis of ribose sugars and NADPH [55]. Aspirin also acetylates other metabolic enzymes such as pyruvate kinase M2 (PKM2) and lactate dehydrogenase (LDH), however, this was not found to affect their enzymatic activity [56]. G6PD is overexpressed in CRC patient specimens and predicts poor prognosis [57], and therefore, suppression of G6PD activity may be an important mechanism underlying the anti-tumour effects of aspirin.

Some recent studies have suggested that the growth-inhibitory effects of aspirin may be dependent on targeting glutamine metabolism. Glutamine deprivation was found to limit the anti-growth effect of aspirin in HCT-15 and HCT116 cells [CRC cell lines harbouring phosphatidylinositol-4,5-bisphosphate 3-kinase catalytic subunit alpha (PIK3CA) mutations] [58]. Interestingly, a number of studies have linked the efficacy of aspirin to PIK3CA mutational status [59, 60]; evidence has shown that aspirin inhibits the phosphorylation and therefore activation of PI3K and Akt [59]. Aspirin treatment appears to mimic the effects of glutamine depletion on the cell cycle and biosynthetic pathways, by both inducing G1 arrest and causing mTOR complex 1 (mTORC1) inhibition [58]. Consistent with this, aspirin was shown to upregulate glutaminolysis-associated genes such as GLS 1, alanine-serine-cystine transporter 2 (ASCT2), large neutral amino acid transporter 1 (LAT1), and glutamic-pyruvic transaminase 2 (GPT2) in PIK3CA mutant CRC cells [58]. This upregulation by aspirin is proposed to be via the induction of activating transcription factor 4 (ATF4) signalling, a major metabolic regulator often upregulated in response to glutamine deprivation [61].

In support of this work, aspirin was shown to regulate cellular metabolism in CRC cells but this was not dependent on PIK3CA mutational status [62]. Aspirin was found that aspirin downregulates a number of key glutaminolysis enzymes including GPT2, glutamate dehydrogenase 1 (GLUD1), glutamic-oxaloacetic transaminase 2 (GOT2), and the glutamine transporters *SLC7A11* and *SLC7A5* leading to reduced levels of glutaminolysis [62]. This work was conducted in cells treated with aspirin long-term (across 52 weeks), which may account for the differences with other published studies. As a likely compensatory mechanism (and consistent with previous work), it was found that CRC cells upregulate GLS1 upon aspirin exposure [62]. This upregulation of GLS1 exposed a metabolic vulnerability which was exploited in a treatment combination with telaglenastat (CB-839), a potent and selective inhibitor of GLS1 [63]. CB-839 is in phase I clinical trials in patients with solid tumours (ClinicalTrials.gov identifier: NCT02071862) and advanced CRC (ClinicalTrials.gov identifier: NCT02861300) but has shown limited efficacy when used as a monotherapy. It was demonstrated that both long-term (52 weeks) and short-term (72 h) aspirin treatment sensitised CRC cell lines to CB-839. Importantly, the combination of aspirin and CB-839 was shown to reduce colon crypt proliferation *in vivo* [62]. Excitingly, these findings add weight to the growing evidence that aspirin has therapeutic potential, in addition to chemoprevention, as an adjuvant to metabolic inhibitors currently under clinical investigation.

## Aspirin and the key drivers of malignant cell metabolism

**Figure 3:**
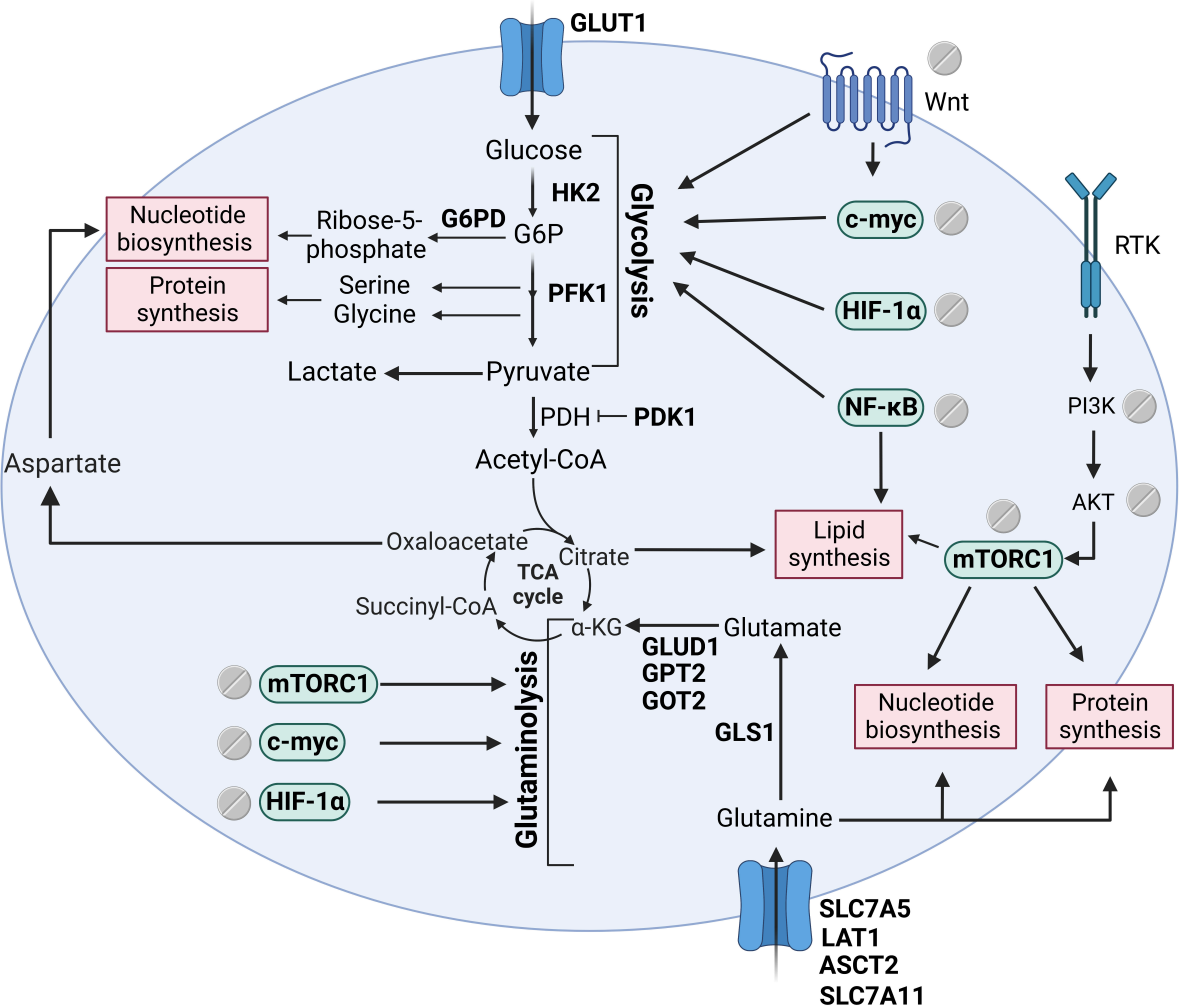
Signalling pathways that support tumour biomass production regulated by aspirin. Tumour cells frequently have aberrant activation of key signalling pathways such as the Wnt, mTOR, NF-κB, and HIF-1α. Aspirin has been shown to regulate these important oncogenic drivers and a number of downstream metabolic enzymes leading to a profound effect on metabolic reprogramming. The figure includes metabolic enzymes regulated by aspirin (in bold), signalling pathways regulated by aspirin and their downstream effect on metabolism, and key biosynthetic branchpoints. The figure was created with BioRender.com. HK2: hexokinase 2; G6P: glucose-6-phosphate; PFK1: phosphofructokinase 1; PDH: pyruvate dehydrogenase; PDK1: PDH kinase 1; α-KG: α-ketoglutarate

As mentioned above, oncogenic pathways frequently dysregulated in CRC such as Wnt, NF-κB, mTOR, and HIF-1α are reported to be targeted by aspirin. These key signalling nodes also drive metabolic reprogramming in tumour cells suggesting that the regulation of these pathways by aspirin may be key to its metabolic effect (summarised in **Figure 3**).

### HIF pathway

Hypoxia is a common feature of solid tumours; chronic proliferation means tumours quickly outgrow their blood supply and the tumour core often has poor perfusion from the vasculature. To survive in harsh environments where oxygen levels are low, tumour cells must rewire cellular metabolism to favour glycolysis and therefore promote the Warburg effect. This metabolic reprogramming is driven by the transcription factor HIF-1α. Under hypoxic conditions, HIF-1α increases the expression of genes encoding glycolytic enzymes to promote glucose utilisation and decrease oxygen consumption which also decreases mitochondrial ROS production. For example, HIF-1α increases the expression of PDK1 to increase pyruvate to lactate conversion and prevent carbon entry into the TCA cycle, attenuating hypoxia-driven ROS production [64, 65]. To ensure biosynthetic demands are met, HIF-1α-driven metabolic reprogramming increases glucose uptake and subsequently glycolytic flux via upregulating the GLUT1 (encoded by *SLC2A1*) and GLUT3 (encoded by *SLC2A3*), as well as hexokinases (HK1 and HK2), enolases (ENOs, ENO1 and ENO2), PKM, and LDHA [66–69]. HIF-1α also increases the expression of glutamine transporters and GLSs to increase intracellular glutamate levels and glutathione levels to protect against ROS [70–72]. Additionally, HIF-1α has been demonstrated to promote acetyl-CoA production from glutamine to fuel lipogenesis [73].

A number of studies have highlighted the role of aspirin in glucose metabolism, proposing that its effect is mediated through HIF-1α, which may contribute to its anti-tumour activity. The impact of aspirin on glucose metabolism was first observed in a study on human platelets; incubation with aspirin markedly inhibited glycolysis and reduced ATP levels [74]. With respect to cancer, aspirin has been shown to decrease levels of HIF-1α in hepatocellular carcinoma leading to a downregulation of GLUT1, decreased glucose import, and reduced cellular proliferation [75]. Aspirin has also been shown to decrease expression of *H19*, which sequesters the HIF-1α negative regulator let-7, thereby reducing HIF-1α levels [76]. Aspirin has been shown to suppress PDK1 expression in breast cancer stem cells in a dose-dependent manner which authors suggested to be via a HIF-1α signalling axis [76]. PFK is a rate-limiting glycolytic enzyme and HIF-1α target which catalyses the conversion of fructose-1-phosphate to fructose-1,6-biphosphate. PFK is highly regulated by allosteric activators such as adenosine monophosphate (AMP) and ADP and inhibitors such as ATP and citrate and is a key mediator of glycolytic flux. Aspirin, and its active metabolite salicylic acid, diminished PFK activity in MCF-7 breast cancer cells, diminishing glycolytic rate and cell viability, although high concentrations of aspirin were used in this study (up to 10 mmol/L) [77]. In sorafenib-resistant hepatocellular carcinoma cells, the combination of sorafenib and aspirin treatment induced tumour cell death both *in vitro* and *in vivo* [78]. Sorafenib resistance was overcome by aspirin via the downregulation of PFK and subsequently glycolysis. This was purported to be via the HIF-1α pathway [78] and is an example of aspirin as a possible therapeutic adjunct in cancer treatment. HK2, another glycolytic target of HIF-1α, was found to be overexpressed in BReast CAncer 1 (BRCA1) mutant ovarian cancers. Aspirin reduced HK2 expression in a dose-dependent manner subsequently reducing glycolytic rate [79], the authors suggest that aspirin may be a promising adjuvant therapy for BRCA1 mutant ovarian cancer patients.

Although there is limited evidence of an association between HIF-1α and aspirin in CRC specifically, HIF-1α is commonly overexpressed in CRC and has been found to be associated with poorer prognosis [80]. CRC cells have been shown to exhibit increased glucose consumption and lactate excretion often via the regulation of glycolytic enzymes such as GLUT1, HK2, PK, PDK1, and LDH [81–85], all of which are HIF-1α targets. It is interesting to speculate that the regulation of these enzymes by aspirin is in part due to the HIF-1α signalling axis.

### NF-κB pathway

NF-κB is a family of transcription factors best known for inducing the expression of genes involved in cell survival and inflammation. Elevated NF-κB activity is frequently observed in a variety of cancers including CRC where it has a key role in cell proliferation and survival and has been associated with late-stage CRC and worse overall survival [86–88]. In addition to its more established role, there is also evidence for the role of NF-κB signalling in energy homeostasis and cellular metabolism. NF-κB regulates the balance between glycolysis and mitochondrial metabolism, promoting increased glycolysis through the regulation of HK2 or GLUT3 [89, 90]. As NF-κB signalling is induced under stress conditions, it is not surprising that NF-κB mediates metabolic reprogramming. High glucose levels stimulate the inhibitor of nuclear factor-kappa B kinase (IKK)/NF-κB activity; this positive feedback loop is thought to be due to *O*-GlcNAcetlyation of IKKβ and promotes sustained activation of NF-κB subunits [91]. Additionally, constitutive IKKβ activation in hepatocytes gives rise to elevated rates of lipogenesis [92].

There is evidence that aspirin may mediate metabolic reprogramming via the regulation of NF-κB. GLUT1 expression at messenger RNA (mRNA) and protein levels in vascular endothelial cells was shown to be significantly downregulated by 4 mmol/L aspirin [93]. This downregulation of GLUT1 led to decreased glucose import and lactate generation, antagonising the Warburg effect. This study also observed a decrease in phosphorylated p65, consistent with aspirin regulating its activation [93]. NF-κB signalling was shown to drive a metabolic programme favouring glycolysis via the upregulation of GLUT1 which may in part account for the downregulation of GLUT1 in response to aspirin [94]. This has been observed in hepatoma cells and an NF-κB regulatory binding element was identified within the GLUT1 gene promoter [75]. Aspirin also affects NF-κB signalling which has been shown to activate GLS1 expression in hepatocellular carcinoma [95].

Aspirin has been demonstrated to downregulate 5-FU induced NF-κB signalling in resistant SW480 and SW620 CRC cell lines which sensitised cells to 5-FU and inhibited growth [96]. This increased 5-FU efficacy was shown both *in vitro* and *in vivo* [96], making aspirin a promising adjuvant therapy in CRC. The effect of aspirin on NF-κB signalling and its downstream consequences on cellular metabolism may be a key driver of CRC cell metabolic reprogramming seen upon aspirin exposure.

### mTOR and AMPK pathway

The mTOR is a kinase complex involved in cellular survival and proliferation. There are subtypes of mTOR; mTORC1 regulates metabolism and biogenesis including protein, lipid, and nucleotide synthesis [97]. mTORC1 is also involved in the shift in metabolism from oxidative phosphorylation to glycolysis (the Warburg effect) in cancer cells [98]. The mTOR pathway is complex and is comprehensively reviewed elsewhere [99]. Briefly, mTORC1 activity is regulated mainly by the PI3K/Akt signalling axis and the adenosine monophosphate-activated protein kinase (AMPK). AMPK is a critical energy sensor that monitors the ratio of AMP:ATP; mTORC1 and AMPK1 work together to couple nutrient availability to metabolism. If the cells are under nutrient deplete conditions, the levels of ATP will be decreased. Excessive AMP activates AMPK1 and causes an increase in catabolic processes such as fatty acid oxidation and a reduction in anabolic processes via the inhibition of mTORC1. The mTOR pathway coordinates metabolic reprogramming in response to nutrient availability.

In 5 mmol/L aspirin-treated CRC cells, aspirin has been shown to reduce mTOR signalling by inhibiting phosphorylation and therefore activation of the downstream effectors ribosomal protein S6 kinase-1 (S6K1) and eukaryotic initiation factor 4E-binding protein 1 (4E-BP1) which are important for protein synthesis [100]. Aspirin also altered the AMP:ATP ratio which in turn activated AMPK and subsequently inhibited mTORC1. This was also demonstrated *in vivo*, patients given an aspirin dose of 600 mg/day for seven days had a reduced phosphorylation status of S6K1 and S6 (downstream targets of mTORC1) in their rectal mucosa [100]. This inhibition of mTOR by aspirin was also seen in PIK3CA mutant breast cancer cells [101]. The effect of aspirin on mTOR signalling and the consequence on cellular metabolism and biosynthesis may prove to be a key mechanism of chemoprevention against CRC.

### Wnt pathway

Wnt signalling plays a key etiological role in the progression of CRC and has been shown to influence metabolic rewiring. Metabolic reprogramming mediated by Wnt favours aerobic glycolysis, in part due to the upregulation of PDK1 which is required for the shift away from mitochondrial respiration [102]. Wnt signalling also regulates the expression of PKM2 and LDHA [103], both of which are involved in the promotion of the Warburg effect. c-myc is an important transcription factor downstream of Wnt and is a metabolic regulator. In triple-negative breast cancer, Wnt5B was shown to suppress mitochondrial function through the c-myc signalling axis [104]. c-myc controls the transcription of a large number of genes involved in glucose metabolism such as LDH, GLUT1, HK2, and PFK contributing to increased glucose consumption and lactate synthesis [105]. c-myc is also involved in glutamine metabolism by upregulating the glutamine transporters *SLC7A5* and *SLC1A5* and promoting glutaminolysis via transcriptional repression of the GLS1 negative regulator miRNA-23a/b (miR-23a/b) [106–108]. c-myc also induces glutamine synthetase expression which catalyses the conversion of glutamate and ammonia to glutamine [109].

Aspirin treatment has been shown to decrease the nuclear pool of β-catenin in CRC lines [110]. This was achieved by phosphorylation and inhibition of protein phosphatase 2A (PP2A) which is a positive regulator of β-catenin. Aspirin reverses the Wnt-mediated cystic phenotype in intestinal organoids by decreasing Wnt signalling and stemness marker expression [111]. Furthermore, it was demonstrated that aspirin reduced c-myc expression in HCT-116 CRC cells [56]. In CRC cell lines, aspirin has been shown to decrease glutamine transporter expression and decrease glutamine metabolism [58, 62], which may be in part due to c-myc downregulation. This highlights aspirin’s important role in targeting this frequently dysregulated pathway in CRC and its metabolic consequences.

## Conclusions

This review focuses on the emerging role of aspirin as a regulator of metabolic reprogramming. Cancer cells frequently undergo metabolic rewiring, driven by oncogenic pathways such as HIF, Wnt, mTOR, and NF-κB, to support the increased proliferative rate as tumours develop and progress. Although specific metabolic inhibitors have gained momentum for cancer therapy, their use has often proved ineffective due to the metabolic plasticity of cancer cells. Cellular metabolic reprogramming has been identified as a key mechanism of action of aspirin and includes the regulation of key metabolic drivers, glycolytic and glutaminolysis enzymes, and altered nutrient utilisation upon aspirin exposure. The targeting of metabolic plasticity by aspirin exposes metabolic vulnerabilities and provides an exciting opportunity for the use of aspirin in combination with specific metabolic inhibitors (including GLS inhibitors such as CB839 and IACS-6274, currently in clinical trials) for the treatment of CRC and potentially other cancers. Although there remains debate around the optimal dose and duration of aspirin treatment to minimise risk in the context of prevention [6], research also highlights a new role for aspirin in improving the efficacy of a new generation of metabolic inhibitors currently undergoing clinical investigation.

## Abbreviations

5-FU: 5-fluorouracil
Akt: protein kinase B or Akt
AMP: adenosine monophosphate
AMPK: adenosine monophosphate-activated protein kinase
ATP: adenosine triphosphate
CB-839: telaglenastat
c-myc: cellular myc
COXs: cyclooxygenases
CRC: colorectal cancer
ENOs: enolases
G6PD: glucose-6-phosphate dehydrogenase
GI: gastrointestinal
GLS: glutaminase
GLUT1: glucose transporter 1
GPT2: glutamic-pyruvic transaminase 2
HIF: hypoxia-inducible factor
HK2: hexokinase 2
IKK: inhibitor of nuclear factor-kappa B kinase
LDH: lactate dehydrogenase
mTOR: mammalian target of rapamycin
mTORC1: mammalian target of rapamycin complex 1
NF-κB: nuclear factor kappa light chain enhancer of activated B cells
NSAID: nonsteroidal anti-inflammatory drug
PDK1: pyruvate dehydrogenase kinase 1
PFK1: phosphofructokinase 1
PI3K: phosphatidylinositol 3-kinase
PIK3CA: phosphatidylinositol-4,5-bisphosphate 3-kinase catalytic subunit alpha
PKM2: pyruvate kinase M2
ROS: reactive oxygen species
TCA: tricarboxylic acid
Wnt: wingless-related integration site

## Declarations

### Copyright

© The Author(s) 2023.
